# Supplementary material for: Ni-Catalyzed Enantioselective Intramolecular Mizoroki–Heck Reaction for the Synthesis of Phenanthridinone Derivatives
Source: J Org Chem. 2023 Jun 15;88(13):8203–26. doi: 10.1021/acs.joc.3c00202 (PMC10337041; doi:10.1021/acs.joc.3c00202)
Supplement: Supplementary file 2 — jo3c00202_si_002.zip [file jo3c00202_si_002.zip › Amides (N-MOM)/1m Me-NMOM-ArBr pyridine C3_N/13C Me-NMOM-pyridine C3_N ArBr 1m/8/pdata/1/email_DR-112-111 F12-20 C13 350 K_8_1.pdf]

DR-112-111 F12-20 HSQC 350 K
HSQCETGP DMSO {D:\nmrusers\malachowski} BM 9

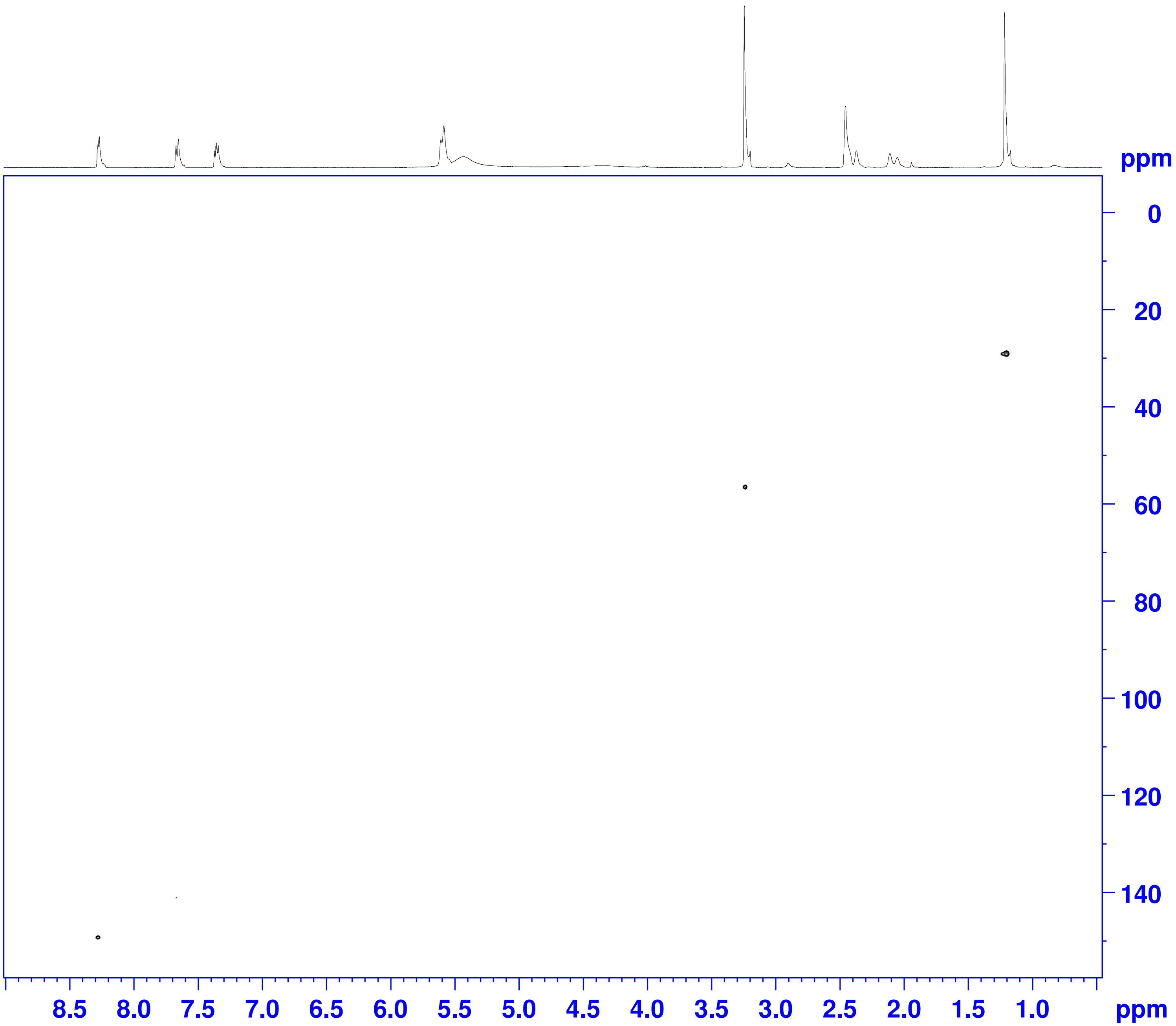

Current Data Parameters

NAME	DR-112-111 F12-20 C13 350 K	
EXPNO	8	
PROCNO	1	

F2 - Acquisition Parameters

Date_	20211130	
Time	21.31	h
INSTRUM	spect	
PROBHD	Z104450_0352 (	
PULPROG	hsqcetgp	
TD	1024	
SOLVENT	DMSO	
NS	2	
DS	16	
SWH	3424.657	Hz
FIDRES	6.688784	Hz
AQ	0.1495040	sec
RG	203	
DW	146.000	usec
DE	6.50	usec
TE	350.1	K
CNST2	145.0000000	
D0	0.00000300	sec
D1	1.50000000	sec
D4	0.00172414	sec
D11	0.03000000	sec
D16	0.00020000	sec
IN0	0.00003010	sec
TDav	1	
ZGOPTNS		
SFO1	400.1519153	MHz
NUC1	1H	
P1	12.75	usec
P2	25.50	usec
P28	1000.00	usec
PLW1	12.14200020	W
SFO2	100.6253439	MHz
NUC2	13C	
CPDPRG[2	garp	
P3	8.00	usec
P4	16.00	usec
PCPD2	80.00	usec
PLW2	56.13299942	W
PLW12	0.56133002	W
GPNAM[1]	SMSQ10.100	
GPZ1	80.00	%
GPNAM[2]	SMSQ10.100	
GPZ2	20.10	%
P16	1000.00	usec

F1 - Acquisition parameters

TD	256	
SFO1	100.6253	MHz
FIDRES	129.775742	Hz
SW	165.081	ppm
FnMODE	Echo-Antiecho	

F2 - Processing parameters

SI	1024	
SF	400.1500201	MHz
WDW	QSINE	
SSB	2	
LB	0	Hz
GB	0	
PC	1.40	

F1 - Processing parameters

SI	1024	
MC2	echo-antiecho	
SF	100.6177975	MHz
WDW	QSINE	
SSB	2	
LB	0	Hz
GB	0	
